# (–)-Epigallocatechin-3-Gallate Ameliorates Atherosclerosis and Modulates Hepatic Lipid Metabolic Gene Expression in Apolipoprotein E Knockout Mice: Involvement of TTC39B

**DOI:** 10.3389/fphar.2018.00195

**Published:** 2018-03-09

**Authors:** Wei Wang, Zheng-Zhu Zhang, Yan Wu, Ru-Qing Wang, Jin-Wu Chen, Jing Chen, Yan Zhang, Ya-Jun Chen, Ming Geng, Zhong-Dong Xu, Min Dai, Jin-Hua Li, Li-Long Pan

**Affiliations:** ^1^School of Life Science, Hefei Normal University, Hefei, China; ^2^State Key Laboratory of Tea Plant Biology and Utilization, Anhui Agricultural University, Hefei, China; ^3^Key Laboratory of Xin’an Medicine, Ministry of Education, Anhui Key Laboratory for Research and Development of Traditional Chinese Medicine, School of Pharmacy, Anhui University of Chinese Medicine, Hefei, China; ^4^School of Medicine, Jiangnan University, Wuxi, China

**Keywords:** (–)-epigallocatechin-3-gallate, tetratricopeptide repeat domain protein 39B, atherosclerosis, inflammation, dyslipidemia

## Abstract

**Background:** Aberrant chronic inflammation and excess accumulation of lipids play a pivotal role in the occurrence and progression of atherosclerosis. (–)-Epigallocatechin-3-gallate (EGCG), the major catechins in green tea, displayed anti-atherosclerotic properties *in vivo* and *in vitro*. However, the effects and underlying mechanism of EGCG on atherosclerosis remain unclear.

**Methods:** Male apolipoprotein E-knockout (ApoE^-/-^) mice (7 weeks old) fed with high-fat diet (HFD) were treated with normal saline or EGCG (40 mg/kg/d, i.g.) for 18 weeks. Atherosclerotic plaque and liver lipid accumulation were measured by Oil Red staining. Plasma lipids and cytokines were detected using commercial kits. The expression of protein and mRNA was analyzed by western blot and quantitative real-time reverse transcription-polymerase chain reaction, respectively.

**Results:** EGCG administration markedly attenuated atherosclerotic plaque formation in HFD-fed ApoE^-/-^ mice, which were accompanied by increased plasma interleukin-10 (IL-10) level and decreased plasma IL-6 and tumor necrosis factor-α (TNF-α) levels. In addition, EGCG modulated high-fat-induced dyslipidemia, evidencing by decreased total cholesterol (TC) and low-density lipoprotein levels and increased high-density lipoprotein level. Meanwhile, EGCG treatment alleviated high-fat-mediated liver lipid accumulation and decreased liver TC and triglyceride. Mechanistically, EGCG significantly modulated high-fat-induced hepatic tetratricopeptide repeat domain protein 39B (TTC39B) expression and its related genes (*Lxrβ, Abcg5, Abcg8, Abca1, Srebf1, Scd1, Scd2, Fas, Elovl5, Mylip*) expression in liver from ApoE^-/-^ mice. Notably, EGCG remarkably induced hepatic liver X receptor α (LXRα) and LXRβ expression and inhibited both precursor and mature sterol regulatory element binding transcription factor-1 (SREBP-1) expression.

**Conclusion:** Taken together, our data for the first time suggested that TTC39B was involved in EGCG-mediated anti-atherosclerotic effects through modulation of LXR/SREBP-1 pathway.

## Introduction

Atherosclerosis is a highly prevalent disease that can significantly increase the risk of major vascular events, such as myocardial or cerebral infarctions (Koon et al., 2011). Atherosclerosis is a multifactorial disease consisting of a multitude of pathogenic developments, including foam cell formation and death, extracellular lipid accumulation, chronic inflammation, and smooth muscle cell proliferation [*reviewed in* Ross (1999) and Pan et al. (2017)]. There is growing evidence that both chronic inflammation and hyperlipidemia are key risk factors for the development of vascular diseases including atherosclerosis [*reviewed in* Back and Hansson (2015)]. Atherosclerotic plaque is usually made of fatty substances (cholesterol, triglycerides, lipoprotein, etc.), it is the main culprit that causes atherosclerosis, and it can partially or completely block the blood flow in the arteries [*reviewed in* Hansson (2009)].

A human genome-wide association study has revealed tetratricopeptide repeat domain protein 39B (TTC39B) gene associated with a change in blood lipoprotein levels (Teslovich et al., 2010). The nuclear receptor liver X receptors (LXRs) upregulated a series of genes, including *Abcg5, Abcg8, Abca1*, and so on, which promote coordinated mobilization of excess cholesterol from cells and from the body [Reviewed in Calkin and Tontonoz (2012)], making it a potentially therapeutic target for the treatment of metabolic and atherosclerotic diseases. Hsieh et al. (2016) elucidated the functional association between TTC39B and LXRs and provided evidence that TTC39B deficiency stabilized endogenous LXRs level and its target genes as well as decreased the incidence of atherosclerosis and fatty liver. Furthermore, TTC39B deficiency also inhibited hepatic sterol regulatory element-binding protein 1 (SREBP-1) (Hsieh et al., 2016), which controls lipogenic gene expression [reviewed in Wang Y. et al. (2015)]. Thus, inhibition of T39, and thus stabilization of endogenous LXRs, provided insights into treating metabolic diseases, including atherosclerosis and fatty liver [reviewed in Loaiza et al. (2017) and Tran and Wang (2017)].

Green tea (*Camellia sinensis*) is an extremely popular beverage worldwide, is next to water, and its habitual consumption has long been associated with health benefits [*reviewed in* Singh et al. (2011)]. Green tea and tea constituents have a potential protective effect against cardiovascular disease that may be due to lowering lipid levels (Ding et al., 2017). Among natural compounds of particular interest, (–)-epigallocatechin-3-gallate (EGCG) has gained significant attention in the past decade for its health benefits. EGCG, a major catechin component of green tea, has recently been confirmed to be beneficial effective cardiovascular diseases, including acute and chronic myocardial infarction (Devika and Stanely Mainzen Prince, 2008; Lin et al., 2016), ischemic stroke (Park et al., 2010), as well as atherosclerosis (Eng et al., 2018). In the clinical atherosclerotic study, EGCG significantly improved endothelial function and improved plasma lipid profile (Widmer et al., 2013). In experimental studies, drinking water supplemented with EGCG evidently inhibited high-fat-diet (HFD)-induced atherosclerosis in ApoE-knockout (ApoE^-/-^) mice (Miura et al., 2001; Yin et al., 2016). The salubrious effects of EGCG may be due to its various biological activities, such as anti-oxidative, anti-inflammatory, and hypolipidemic activities (Eng et al., 2018). However, the exact mechanism of EGCG on atherosclerosis remains unclear.

Therefore, the aim of the present study was to evaluate whether EGCG treatment modulated (1) high-fat-induced atherosclerosis; (2) inflammatory response and lipid profile in experimental atherosclerotic mice; and (3) TTC39B is involved in EGCG-mediated anti-atherosclerotic activities in experimental atherosclerotic mice.

## Materials and Methods

### Chemicals and Reagents

(–)-Epigallocatechin-3-gallate (≥95%) was purchased from Sigma-Aldrich (Sigma Chemical Co., St. Louis, MO, United States). Total cholesterol (TC), triglycerides (TG), high-density lipoprotein-cholesterol (HDL-C), low-density lipoprotein-cholesterol (LDL-C), alanine aminotransferase (ALT), and aspartate aminotransferase (AST) assay kits were purchased from Jiancheng Bio-engineering Institute (Nanjing, China). Trizol reagent was obtained from Invitrogen Inc. (Carlsbad, CA, United States), PrimerScript RT Reagent Kit was purchased from Takara Bio (Shiga, Japan), and the quantitative real-time reverse transcription (RT)-polymerase chain reaction (qPCR) kit was purchased from Bio-Rad Laboratories (Hercules, CA, United States). Antibodies against TTC39B (ab107673), LXRα (ab176323), LXRβ (ab28479), and sterol regulatory element-binding transcription factor 1 (SREBP-1) (ab28481) were purchased from Abcam (Cambridge, United Kingdom). Anti-glyceraldehyde-3-phosphate dehydrogenase (GAPDH, sc-25778) antibody was purchased from Santa Cruz Biotechnology (Santa Cruz, CA, United States).

### Experimental Procedures

Animal experimental protocols used for the study have been approved by the Animal Care and Ethical Use Committee of Hefei Normal University, in accordance to the guidelines for care and use of animals established by the Hefei Normal University. Thirty 7-week-old male ApoE^-/-^ (18–24 g, C57BL/6J background) mice were obtained from Vital River Laboratory Animal Technology Co. Ltd. (Beijing, China). Mice were provided with a standard rodent chow diet and distilled water *ad libitum* and housed at 21 ± 3°C, 60 ± 10% relative humidity, and exposed to 12 h light–12 h dark cycles. After 1-week acclimation, ApoE^-/-^ mice were divided randomly into two groups (*n* = 15 for each group) and were fed with HFD (D12079B, Research Diets, Inc., New Brunswick, NJ, United States^1^) with or without treatment for 18 weeks as follows: (1) ApoE^-/-^/control group was fed a HFD and (2) ApoE^-/-^/treatment group was fed an HFD and administered EGCG (40 mg/kg/d, i.g.). The dose of EGCG (40 mg/kg/d, i.g.) chose is based on our pre-trial and previous studies (Wolfram et al., 2006; Santamarina et al., 2015). EGCG was dissolved in distilled water and a total volume of 0.1 mL was administered daily. The control group was administered with 0.1 mL distilled water. At the end of the experiment, all mice were euthanized after being food-deprived for 12 h. Plasma was prepared by centrifugation within 15 min after collection at 1,550 × *g* for 20 min at 4°C (Allegra X-30R; Beckman Coulter, Inc., Brea, CA, United States). Liver tissues were quickly dissected and weighed (liver coefficient is determined by the ratio of wet liver weight:body weight). The sample of liver was divided into six parts and placed in a 1.5 mL Eppendorf tube. These plasma and liver samples were then stored at -80°C for further analysis. Aortas were collected from the base of ascending aorta and to the iliac bifurcation, whereas aortic roots with heart were harvested and both are fixed in 4% paraformaldehyde.

### Plasma and Hepatic Lipid Parameters

Plasma TG, TC, HDL-C, LDL-C, ALT, and AST concentrations were assayed using common commercially available biochemical kits (JianCheng Bioengineering Institute, Nanjing, China). The lipids were extracted from liver tissues as previously described (Wang W. et al., 2015), and then TC and TG levels were determined using plasma TC and TG determination kits.

### Atherosclerotic Lesion and Liver Tissue Analysis

For *en face* analysis of the atherosclerotic lesions, the entire aorta was isolated and stained with Oil Red O. Briefly, the entire aorta was dissected and opened longitudinally. After staining with Oil Red O (Sigma-Aldrich; Sigma Chemical Co., St. Louis, MO, United States), *en face* images of the aorta were taken with a dissecting microscope (Motic, China) and analyzed using the ImageJ software (version 1.42q, NIH). The lesion area of each mouse is described as the percentage of total luminal surface. Aortic sinuses and liver tissues were embedded in OCT embedding medium, and cryosections (8 μm) were stained with Oil Red O. Mean lesion area was calculated from 14 consecutive Oil Red O-stained sections per mouse. Images of sections were obtained by a light microscope (Olympus BX41, Tokyo, Japan) and the Oil Red O positive atherosclerotic lesion area was measured using the ImageJ software (version 1.42q, NIH).

### Quantitative Real-Time Reverse Transcription-Polymerase Chain Reaction (qPCR)

The mRNA expression levels of *Ttc39b, Lxrα, Lxrβ, Abcg5, Abcg8, Abca1, Srebf1, Scd1, Scd2, Fas, Pnpla3, Acss2, Elovl5, Gpam*, and *Mylip* in liver tissues were detected by qPCR as previously described (Wang et al., 2013; Wang W. et al., 2015). The primer information is given in **Table 1**. Briefly, total RNA from liver tissues was extracted by Trizol and 1 μg of RNA was used to generate cDNA using a PrimerScript RT Reagent Kit (Takara Bio, Shiga, Japan). Reverse transcription (RT) reactions were performed according to the manufacturer’s instructions. The qPCR analysis was performed in triplicate for target mRNAs using a qPCR SYBR Green Mix Kit (Bio-Rad Laboratories, Hercules, CA, United States) and the PCR conditions were as follows: 1 cycle of 95°C, 5 min; 40 cycles of 95°C, 15 s; 60°C, 20 s. The fluorescent signals of SYBR Green were subjected to cDNA analysis using an ABI StepOne machine (Applied Biosystems, Forster, CA, United States). Melting curve analysis was performed to confirm specificity. The 2^-ΔΔC_T_^ method was used for the semi-quantitative PCR analysis. Target RNA levels were normalized to GAPDH mRNA.

**Table 1:** SYBR green primer information.

Gene symbol	Gene name	GenBank Acc. No.	Primer sequences (5′→3′)	*T*_m_ (°C)	*E* (%)	Am (bp)
*Ttc39b*	Tetratricopeptide repeat domain 39B	NM_027238	F: TGAGCCTTTTCAGTGTTCCCT	81.0	95	171
			R: CATCCTGGCCTTCCATACCTA			
*Lxrα*	Nuclear receptor subfamily 1, group H, member 3	AF085745	F: TGACTCCAACCCTATCCCTAA	82.0	91	141
			R: GTTTCTCCTGATTCTGCAACG			
*Lxrβ*	Nuclear receptor subfamily 1, group H, member 2	NM_009473	F: GGCGATAAGCAAGGCATACTC	84.0	92	182
			R: AAACAGCCAGACGCTACAACC			
*Abcg5*	ATP-binding cassette subfamily G member 5	AH011511	F: CATCTGCCACTTATGATACAGG	84.7	94	236
			R: CAGTGATTATGCGTCTCGTTC			
*Abcg8*	ATP-binding cassette subfamily G member 8	AH011518	F: AAGGCTGAGTATCACCAGTCTTGAA	79.4	104	135
			R: GGACCTGAACTCGCATCCACT			
*Abca1*	ATP-binding cassette subfamily A member 1	NM_013454	F: GGCAGCATCTTCTTGATTTTGTC	83.4	94	163
			R: GAAACCCAATCCCAGATACCC			
*Srebf1*	Sterol regulatory element binding transcription factor 1	NM_011480	F: CACTTCGTAGGGTCAGGTTCT	81.4	99	101
			R: GCTGAATAAATCTGCTGTCTTG			
*Scd1*	Stearoyl-Coenzyme A desaturase 1	NM_009127	F: CCGTGCCTTGTAAGTTCTGTG	82.3	93	92
			R: GCCTCTTCGGGATTTTCTACT			
*Scd2*	Stearoyl-Coenzyme A desaturase 2	NM_009128	F: CGAACCTTTACTGTAGACCCTTG	80.5	99	117
			R: TTGTCCCTGATGACCTCGTTT			
*Fas*	Fatty acid synthase	NM_007988	F: CCGTGGTGCTGGAGATTG	85.4	102	168
			R: GGTTGACATTGATGCCTGTGA			
*Pnpla3*	Patatin-like phospholipase domain containing 3	NM_054088	F: CAGGGCAGCATGATGTAGGAC	83.1	92	188
			R: GAAAATGGCAAACTTGTGGGA			
*Acss2*	Acyl-CoA synthetase short-chain family member 2	NM_019811	F: GAAAGTTGTAGCCACATAGAGCATA	83.3	97	231
			R: AGCCAGTCCCCACCAGTTAAG			
*Elovl5*	ELOVL fatty acid elongase 5	NM_134255	F: AACATTCCCTTTCCACAGTCT	78.0	94	115
			R: TCATCAGTTCAAAACCCCTAG			
*Gpam*	Glycerol-3-phosphate acyltransferase, mitochondrial	NM_008149	F: TTCCTTTCCGTCCTGGTGATA	82.8	94	227
			R: GAAGATGAAGACAGTGACTTTGGTG			
*Mylip*	myosin regulatory light-chain interacting protein	NM_153789	F: GTTTCGGTGATGGCTCGGTAG	83.2	91	220
			R: AGAAACTCCTCATTGGGGTCG			
*Gapdh*	Glyceraldehyde-3-phosphate dehydrogenase	NM_001289726	F: AAGAAGGTGGTGAAGCAGG	82.3	92	111
			R: GAAGGTGGAAGAGTGGGAGT			

### Flow Cytometry Analysis

Plasma from the mice were harvested for cytokine measurement using a cytometric bead array (CBA) mouse inflammation kit (BD Biosciences, Cat No. 552364) according to the manufacturer’s guidelines for detecting interleukin-6 (IL-6), IL-10, monocyte chemotactic protein-1 (MCP-1), interferon-γ (IFN-γ), tumor necrosis factor-α (TNF-α), and IL-12 production, simultaneously. The samples were analyzed using a flow cytometer (BD Accuri C6; BD Biosciences, San Jose, CA, United States) and FCAP Array software (BD version 3.1) was used to create the standard curves for each cytokine and convert the mean fluorescence intensity (MFI) values into corresponding concentrations.

### Protein Extraction and Western Blot Analysis

Western blot analyses were performed as previously described (Wang et al., 2013; Wang W. et al., 2015). In brief, liver tissues were homogenized in ice-cold Tris buffer (0.01 M Tris, pH 7.4) and then lysed in extraction buffer with protease and phosphatase inhibitor cocktails (Beyotime Institute of Biotechnology, Haimen, China). Proteins of liver tissues were separated by electrophoresis in a 10% SDS-polyacrylamide gel, and then were transferred onto a PVDF membrane (Millipore, Shanghai, China). Blots were first incubated with an antibody against TTC39B (1:200), LXRα (1:1000), LXRβ (1:1000), SREBP-1 (1:1000), or GAPDH (1:200) overnight at 4°C. After primary antibody incubation, the membranes were washed three times in washing buffer for 10 min each and then incubated with the respective horseradish peroxidase-conjugated secondary antibodies (1:5000; Thermo Fisher Scientific, MA, United States) for 1 h at room temperature. Immunoreactive bands were visualized via the enhanced chemiluminescence (Pierce, Rockford, IL, United States) and quantified via densitometry using ImageJ (version 1.42q, NIH).

### Statistical Analysis

In this study, all statistical analyses were conducted using SPSS 13.0 (SPSS, Chicago, IL, United States) and a two-tailed independent sample *t*-tests or a Mann–Whitney *U*-test as indicated for the comparison of the two groups. Experimental data are expressed as the means ± SEM, and *P* < 0.05 was considered statistically significant.

## Results

### EGCG Attenuated Atherosclerotic Plaque Development in ApoE^-/-^ Mice

**FIGURE 1:**
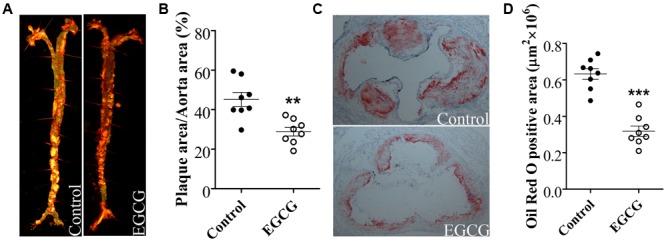
EGCG attenuated atherosclerotic plaque development in ApoE^-/-^ mice. Illustration of ApoE^-/-^ mice were fed with HFD and were treated daily with or without EGCG for 18 weeks by oral gavage. Morphology and quantification of atherosclerotic plaques in the whole aorta **(A,B)** and aortic sinus (magnification, ×40) **(C,D)**. ^∗∗^*P* < 0.01, ^∗∗∗^*P* < 0.001 compared with high-fat-fed ApoE^-/-^ mice (*n* = 8).

As shown in **Figure 1A**, *en face* analysis revealed that atherosclerotic plaques (Oil Red O-stained red area) in the artery formed was observed in high-fat-fed ApoE^-/-^ mice. Intriguingly, EGCG treatment with 40 mg/kg/d significantly reduced the size of Oil Red O-stained atherosclerotic plaques in the aortas (**Figure 1B**). Atherosclerotic burden was further evaluated in cross-sections of the aortic root. Treatment with EGCG (40 mg/kg/d) significantly decreased the lesion area in the aortic root (**Figures 1C,D**). During the entire experimental period, no mice died (data not shown).

### EGCG Altered Plasma Lipid Profile and Hepatic Lipid Metabolism in ApoE^-^*^/^*^-^ Mice

**FIGURE 2:**
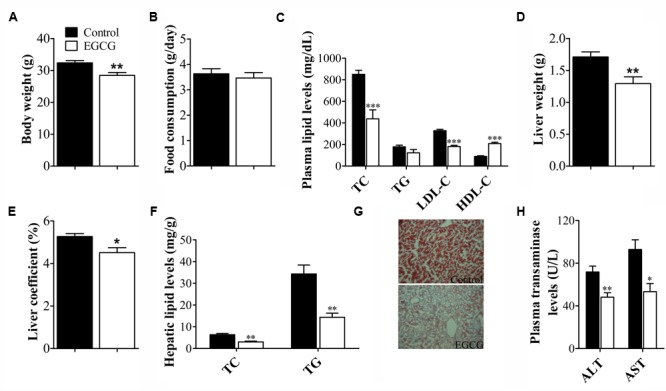
EGCG altered plasma lipid profile and hepatic lipid metabolism in ApoE^-^*^/^*^-^ mice. ApoE^-/-^ mice were fed with HFD and were treated daily with or without EGCG for 18 weeks by oral gavage, body weights, plasma lipid profile, and transaminase levels, and hepatic lipid accumulation was analyzed as described in the section “Materials and Methods.” **(A)** Bar graph showed the quantitative analysis of body weights (*n* = 15). **(B)** Bar graph showed the quantitative analysis of food consumption (*n* = 15). **(C)** Bar graph showed the quantitative analysis of TC, TG, LDL-C, and HDL-C levels in plasma (*n* = 15). Bar graph showed the quantitative analysis of liver weight **(D)** and liver coefficient **(E)** (*n* = 15). **(F)** Quantification of hepatic TC and TG levels (*n* = 15). **(G)** Morphology of hepatic lipid accumulation (magnification, ×200, *n* = 6). **(H)** Quantification of plasma AST and ALT activities (*n* = 15). ^∗^*P* < 0.05, ^∗∗^*P* < 0.01, ^∗∗∗^*P* < 0.001 compared with high-fat-fed ApoE^-^*^/^*^-^ mice.

ApoE^-/-^ mice develop hypercholesterolemia and complex atherosclerotic plaques that closely mimic human lesions. Initial body weights were similar between vehicle- and EGCG-treated ApoE**^-/-^** mice (data not shown), whereas treatment with EGCG for 18 weeks observed lower body weights compared to that of ApoE**^-/-^** mice (**Figure 2A**). There were no statistically significant differences in food intake between the two groups (**Figure 2B**). Meanwhile, plasma lipid profiles were determined in ApoE**^-/-^** mice at the end of 18-week feeding period. As expected, EGCG treatment also dramatically increased HDL-C levels and decreased TC and LDL-C levels in HFD-fed ApoE^-/-^ mice, but has little effect on TG (**Figure 2C**). The liver is the main organ for lipid metabolism. Because EGCG treatment improved the plasma lipid profile, we next determined whether EGCG administration affected the hepatic lipid metabolism. The results showed that compared with control ApoE**^-/-^** mice, supplemented with EGCG significantly decreased liver weight (**Figure 2D**), liver coefficient (**Figure 2E**), TC, and TG content in the liver (**Figure 2F**). The similar profile was further confirmed by Oil Red O staining that EGCG significantly inhibited high-fat-fed-induced hepatic lipid accumulation (**Figure 2G**). Furthermore, EGCG administration also markedly attenuated HFD-induced liver injury in mice (ALT/AST) (**Figure 2H**). These results indicated that EGCG supplementation modulated the lipid disorders in HFD-fed ApoE**^-/-^** mice.

### EGCG Modulated TTC39B Expression and Its Target Gene in ApoE^-^*^/^*^-^ Mice

**FIGURE 3:**
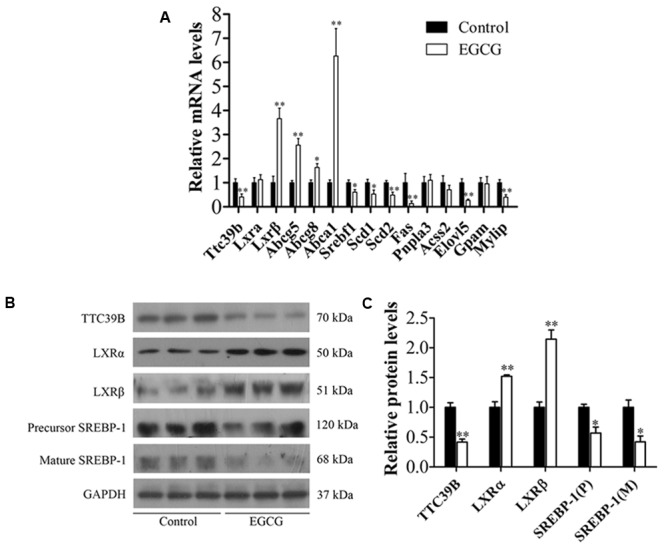
EGCG modulated TTC39B expression and its target gene in ApoE^-^*^/^*^-^ mice. ApoE^-/-^ mice were fed with HFD and were treated daily with or without EGCG for 18 weeks by oral gavage, the protein, or/and mRNA levels of TTC3B, and target genes in liver were detected as described in the section “Materials and Methods.” **(A)** Quantitative analysis of mRNA levels of hepatic *Ttc39b, Lxrα, Lxrβ, Abcg5, Abcg8, Abca1, Srebf1, Scd1, Scd2, Fas, Pnpla3, Acss2, Elovl5, Gpam*, and *Mylip* (*n* = 10). Mann–Whitney *U*-test, ^∗^*P* < 0.05, ^∗∗^*P* < 0.01 compared with high-fat-fed ApoE^-^*^/^*^-^ mice. **(B)** Representative bands of hepatic TTC39B, LXRα, LXRβ, precursor, and mature SREBP-1 protein expression (*n* = 3). **(C)** Quantitative analysis of hepatic TTC39B, LXRα, LXRβ, precursor, and mature SREBP-1 protein levels (*n* = 3). ^∗^*P* < 0.05, ^∗∗^*P* < 0.01 compared with high-fat-fed ApoE^-^*^/^*^-^ mice. See **Supplementary Figure S1** for original images.

To further explore the molecular mechanisms by which EGCG exerted anti-atherosclerotic activity, we examined the effects of EGCG on hepatic TTC39B expression, which modulated lipid metabolism and reduced the process of atherosclerosis (Hsieh et al., 2016). In this experiment, the mRNA and protein levels of hepatic TTC39B in atherosclerotic mice were analyzed by qRCR and western blot, respectively. Intriguingly, treatment with EGCG (40 mg/kg/d) significantly decreased the mRNA and protein levels of hepatic TTC39B in atherosclerotic mice (**Figures 3A–C**). In addition, EGCG administration also markedly increased the expression of hepatic LXRβ and inhibited hepatic SREBP-1 expression at both protein and mRNA levels, but only increased LXRα expression at protein level in high-fat-fed ApoE**^-/-^** mice. Moreover, a decrease of mature SREBP-1 expression was also observed in EGCG-treated ApoE**^-/-^** mice. We subsequently examined the effect of EGCG in LXR target gene expression in hepatic tissue by qPCR. As shown in **Figure 3A**, EGCG treatment significantly induced *Abcg5, Abcg8*, and *Abca1* mRNA levels and decreased the *Scd1, Scd2, Fas, Elovl5*, and *Mylip* mRNA levels. However, there was no changes in *Pnpla3, Acss2*, and *Gpam* mRNA levels following EGCG treatment (**Figure 3A**).

### EGCG Suppressed Systemic Inflammation in ApoE^-^*^/^*^-^ Mice

**FIGURE 4:**
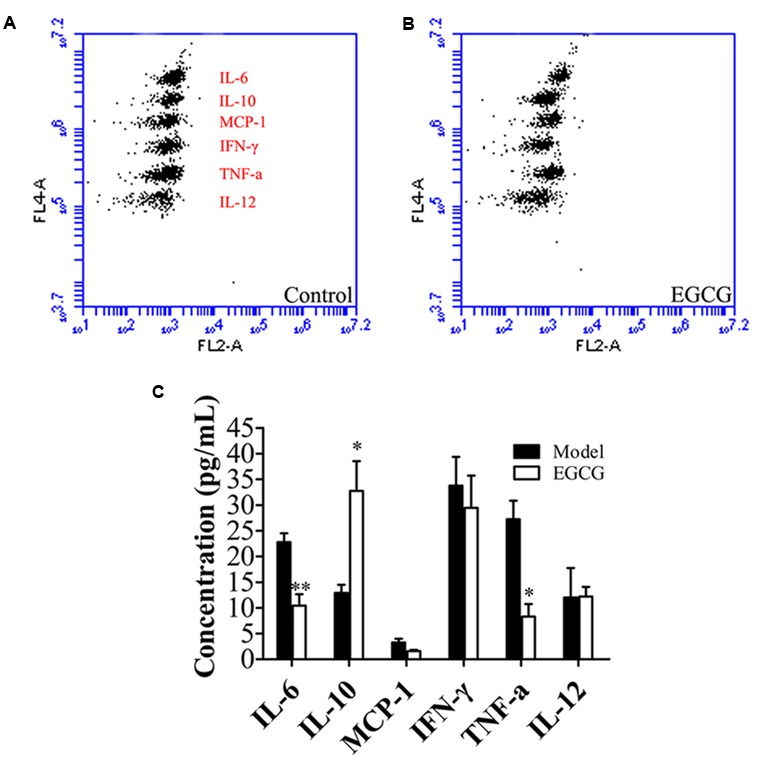
EGCG suppressed systemic inflammation in ApoE^-^*^/^*^-^ mice. ApoE^-/-^ mice were fed with HFD and were treated daily with or without EGCG for 18 weeks by oral gavage, plasma cytokines were detected as described in the section “Materials and Methods.” **(A,B)** Representative flow cytometry of plasma cytokines. **(C)** Quantification of levels of plasma cytokines. ^∗^*P* < 0.05, ^∗∗^*P* < 0.01 compared with high-fat-fed ApoE^-/-^ mice (*n* = 15).

In addition to modulating lipid metabolism, activation of LXR has been demonstrated to inhibit the inflammatory response in ApoE**^-/-^** mice (Bradley et al., 2007; Hong et al., 2012). Next, we explored the effects of EGCG treatment on chronic systemic inflammation by using a CBA. The results revealed that among the six examined cytokines (TNF-α, MCP-1, IFN-γ, IL-6, IL-12, and IL-10), EGCG markedly reduced the production of TNF-α and IL-6 and increased the level of IL-10, whereas the concentrations of the other three cytokines were not significantly influenced by EGCG (**Figure 4**).

## Discussion

Historically, consumption of green tea has been associated with health benefits against multiple cardiovascular diseases, including atherosclerosis (Babu et al., 2012; Kawada, 2016; Liu et al., 2016). In the present study, we uncovered a novel mechanism by which EGCG, a major ingredient of green tea, attenuated atherosclerotic plaque formation by modulated system inflammation and dyslipidemia possibly in a TTC39B-dependent manner.

Dyslipidemia, associated with atherosclerosis, consists of a reduction of HDL-cholesterol and an increase in plasma LDL and TG [reviewed in Eckel et al. (2010)]. EGCG is a polyphenolic compound abundant in green tea and several studies reported the relationship between EGCG consumption and the level of blood lipoprotein [reviewed in Legeay et al. (2015)]. For instance, EGCG treatment reduced serum concentrations of TC and TG in *db*/*db* mice or high-fat diabetic mice (Babu et al., 2012; Pathak et al., 2018). Consistent with previous reports, we found that EGCG significantly modulated plasma lipid disorder and hepatic lipid accumulation, which accompanied by reduction of atherosclerotic plaque in high-fat-fed ApoE^-/-^ mice. However, supplemented with EGCG (10 mg/kg) through daily intraperitoneal injections significantly reduced aortic plaque formation in hypercholesterolemic ApoE^-/-^ mice, but no difference was observed in plasma TC level (Chyu et al., 2004). Similarly, drinking water supplemented with green tea extract attenuated the development of atherosclerosis without changing the plasma TC or TG level in ApoE^-/-^ mice (Miura et al., 2001). Different drug delivery and higher dose used may explain the different hypolipidemic effects observed. Firstly, we chose to use EGCG (40 mg/kg) through daily gavage instead of intraperitoneal injections or administration in the drinking water. In addition, when it is orally administrated, EGCG is mainly absorbed in intestine, and gut microbiota plays a pivotal role in its metabolism prior to absorption and is essential for the absorption of its metabolites (such as gallic acid and epigallocatechin) [reviewed in Gan et al. (2016)]. EGCG supplementation prevented HFD-induced alterations in gut microbiota, such as *Firmicutes/Bacteroidetes* ratio and microbial diversity (Remely et al., 2017). Changes in gut microbiota composition due to different dietary feeding might be relevant for several chronic conditions, including atherosclerosis (Ryan et al., 2017). Finally, EGCG administration in drinking water was easily oxidized and unstable for 24 h (Miura et al., 2001). TTC39B is a novel potential therapeutic target for treating both steatohepatitis and atherosclerosis (Hsieh et al., 2016). TTC39B deficiency displayed increased HDL-cholesterol levels associated with increased enterocyte *Abca1* expression, increased LXR protein, decreased fatty liver, decreased LDL levels, and reduced atherosclerosis (Hsieh et al., 2016). Thus, the study led us to hypothesize that EGCG attenuated high-fat-mediated atherosclerosis at least partially through modulation of TTC39B, an HDL gene discovered in human genome-wide association studies (Teslovich et al., 2010). In support of this, we found that EGCG administration significantly reduced hepatic TTC39B expression at both mRNA and protein levels in high-fat-fed ApoE^-/-^ mice. LXRs are oxysterol-activated nuclear receptors and the primary function of LXRs is thought to be cholesterol homeostasis through upregulation of target genes, including *Abcg5, Abcg8, Abca1*, and so on, which mediate cellular cholesterol efflux [reviewed in Fessler (2018)]. Due to its ability to enhance ABCA1-dependent reverse cholesterol transport, LXR is an attractive target for the treatment of atherosclerosis (Kim et al., 2015). TTC39B can promote the ubiquitination and degradation of LXR. Conversely, TTC39B deficiency stabilizes LXR protein without change in LXR mRNA (Hsieh et al., 2016). Consistent with these findings, EGCG-treated ApoE^-/-^ mice displayed decreased TTC39B levels at both mRNA and protein levels associated with increased hepatic LXRβ at both mRNA and protein levels and increased LXRα protein expression, accompanied by reduction of hepatic lipid accumulation and atherosclerotic plaque. However, the precise mechanism of EGCG on hepatic LXRs mRNA expression remains to be clarified. At the same time, SREBP-1 is the master regulator of *de novo* fatty acid biosynthesis and implicated in hepatic steatosis through upregulation of expression of many lipogenic genes, including *Scd1* and *Elovl5*, leading to elevated plasma and liver TC levels (Kim et al., 2015). In the present study, EGCG-treated ApoE^-/-^ mice also had significant reductions in hepatic precursor and mature of SREBP-1 expression and *Srebf1* mRNA expression, and this effect was associated with a decrease of lipogenic gene *Scd1, Scd2, Fas, Elovl5*, and *Mylip* mRNA expression. In addition, LXRα-dependent increase in phosphatidylcholine synthesis and incorporation of polyunsaturated fatty acids into multiple phospholipid species which further inhibited hepatic SREBP-1 expression (Hsieh et al., 2016). Collectively, our data suggested that the downregulation of TTC39B by EGCG preserved LXRα and LXRβ expression associated with downregulation of SREBP-1 expression, eventually activated a beneficial profile of gene expression that promotes hypolipidemic effects. However, the precise mechanisms of TTC39B in EGCG-mediated vasculoprotection has not been fully elucidated. This mechanism could be particularly valuable to clarify the role of EGCG in modulation of dyslipidemia.

Vascular chronic inflammation is one of the key early events in the pathogenesis of atherosclerosis (Liu et al., 2012; Back and Hansson, 2015). Dietary supplementation of EGCG counteracted several adverse effects associated with vascular inflammation and atherosclerosis, including decreased secretion of inflammatory cytokines such as IL-6 and TNF-α (Babu et al., 2012; Riegsecker et al., 2013). In addition, EGCG attenuated inflammation, lipid uptake, and intracellular cholesterol accumulation in macrophages (Chen et al., 2017; Wang et al., 2017). Therefore, antioxidant and anti-inflammatory activities of EGCG were responsible for its protective effects against atherosclerosis (Chyu et al., 2004; Devika and Stanely Mainzen Prince, 2008; Singh et al., 2011; Lin et al., 2016; Chen et al., 2017). Consistent with previous reports (Chyu et al., 2004), high-fat-fed ApoE^-/-^ mice presented with both significantly smaller aortic atheromatous area and aortic sinus plaque when treatment with EGCG through intragastric administration. In addition, EGCG also significantly inhibited high-fat-mediated system inflammation, evidencing by the decrease of IL-6 and TNF-α and increase of IL-10. Increased IL-10 levels may create an anti-inflammatory environment and lead to protection against atherosclerosis development (McCarthy et al., 2013; Thompson et al., 2017). We chose to use EGCG (daily 40 mg/kg) intragastric administration, which ensure that the plasma level of EGCG was adequate (Miura et al., 2001; Chyu et al., 2004). With the amount of EGCG administered, there were significantly higher anti-inflammatory and anti-atherogenic capacities both in the systemic circulation and in local vascular tissue. Mechanistically, activation of LXRs upregulates a suite of genes that promote coordinated mobilization of excess cholesterol from cells and from the body [reviewed in Calkin and Tontonoz (2012)]. Moreover, LXRs, like other nuclear receptors, are anti-inflammatory, inhibiting signal-dependent induction of pro-inflammatory genes by nuclear factor-κB, activating protein-1, and other transcription factors [reviewed in Fessler (2018)]. Consistent with this notion, EGCG treatment significantly reduced TTC39B expression and enhanced LXRα expression, with that of *Abca1* and *Abcg5/8* also being increased. Activation of the LXRα-ABCA1/ABCG5/8 pathway exerted anti-inflammatory and lipid-regulatory effects during atherosclerosis [reviewed in Fessler (2018)]. Therefore, reduction of TTC39B and enhancement of the LXRα-ABCA1/ABCG5/8 pathway was at least partly responsible for the inhibitory effect of EGCG on the anti-inflammatory and lipid-regulatory effects in high-fat-induced atherosclerosis.

The major new finding of the present study was that EGCG, a major ingredient of green tea, modulated high-fat-mediated hepatic TTC39B expression, which were responsible for lipid metabolism disorder in ApoE^-/-^ mice. Meanwhile, our results also demonstrated that EGCG administration attenuated the formation of atherosclerotic plaque and system chronic inflammatory processes in atherosclerotic mice. Therefore, our data suggested that EGCG ingestion might have therapeutic potential in the prevention of atherosclerosis.

## Author Contributions

WW and Z-ZZ designed the experiments. WW, YW, R-QW, JC, J-WC, YZ, Y-JC, MG, Z-DX, and L-LP performed the experiments. WW, Z-ZZ, and L-LP performed the data analysis. MD, J-HL, WW, and L-LP wrote the manuscript.

## Conflict of Interest Statement

The authors declare that the research was conducted in the absence of any commercial or financial relationships that could be construed as a potential conflict of interest.

## Footnotes

1 http://www.epsekishin.co.jp/lsg/service/researchdiets/pdf/D12079B.pdf

## Supplementary Material

FIGURE S1 Original bands of Western blot for **Figure 3B**.
