# Corrigendum: (-)-Epigallocatechin-3-Gallate Ameliorates Atherosclerosis and Modulates Hepatic Lipid Metabolic Gene Expression in Apolipoprotein E Knockout Mice: Involvement of TTC39B

**DOI:** 10.3389/fphar.2018.00459

**Published:** 2018-05-01

**Authors:** Wei Wang, Zheng-Zhu Zhang, Yan Wu, Ru-Qing Wang, Jin-Wu Chen, Jing Chen, Yan Zhang, Ya-Jun Chen, Ming Geng, Zhong-Dong Xu, Min Dai, Jin-Hua Li, Li-Long Pan

**Affiliations:** ^1^School of Life Science, Hefei Normal University, Hefei, China; ^2^State Key Laboratory of Tea Plant Biology and Utilization, Anhui Agricultural University, Hefei, China; ^3^Key Laboratory of Xin'an Medicine, Ministry of Education, Anhui Key Laboratory for Research and Development of Traditional Chinese Medicine, School of Pharmacy, Anhui University of Chinese Medicine, Hefei, China; ^4^School of Medicine, Jiangnan University, Wuxi, China

**Keywords:** (-)-Epigallocatechin-3-gallate, tetratricopeptide repeat domain protein 39B, atherosclerosis, inflammation, dyslipidemia

The authorship of this article should be correctly ordered to protect the right ownership of this paper. In addition, Dr. Li-Long Pan has contributed significantly to this study, such that Dr. Pan should be the co-corresponding author in this study. The authorship order should be considered as follows:

Wei Wang^1*^, Zheng-Zhu Zhang^2*^, Yan Wu^1^, Ru-Qing Wang^1^, Jin-Wu Chen^1^, Jing Chen^1^, Yan Zhang^1^, Ya-Jun Chen^1^, Ming Geng^1^, Zhong-Dong Xu^1^, Min Dai^3^, Jin-Hua Li^1^ and Li-Long Pan^4*^

^1^School of Life Science, Hefei Normal University, Hefei, China.

^2^State Key Laboratory of Tea Plant Biology and Utilization, Anhui Agricultural University, Hefei, China.

^3^Key Laboratory of Xin'an Medicine, Ministry of Education, Anhui Key Laboratory for Research and Development of Traditional Chinese Medicine, School of Pharmacy, Anhui University of Chinese Medicine, Hefei, China.

^4^School of Medicine, Jiangnan University, Wuxi, China.

The authors apologize for this error and state that this does not change the scientific conclusions of the article in any way. The original article has been updated.

## Author contributions

WW and Z-ZZ designed the experiments. WW, YW, R-QW, JC, J-WC, YZ, Y-JC, MG, Z-DX, and L-LP performed the experiments. WW, Z-ZZ, and L-LP performed the data analysis. MD, J-HL, WW, and L-LP wrote the manuscript.

### Conflict of interest statement

The authors declare that the research was conducted in the absence of any commercial or financial relationships that could be construed as a potential conflict of interest.

