# Supplementary figures and images for: (–)-Epigallocatechin-3-Gallate Ameliorates Atherosclerosis and Modulates Hepatic Lipid Metabolic Gene Expression in Apolipoprotein E Knockout Mice: Involvement of TTC39B
Source: Front Pharmacol. 2018 Mar 9;9:195. doi: 10.3389/fphar.2018.00195 (PMC5854642; doi:10.3389/fphar.2018.00195)

TTC39B

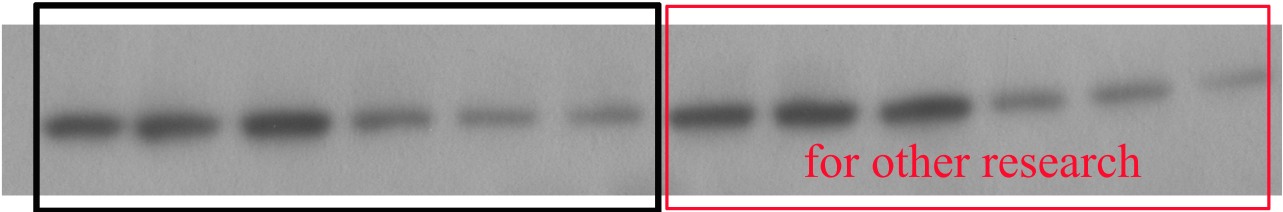

70 kDa

LXR $\alpha$

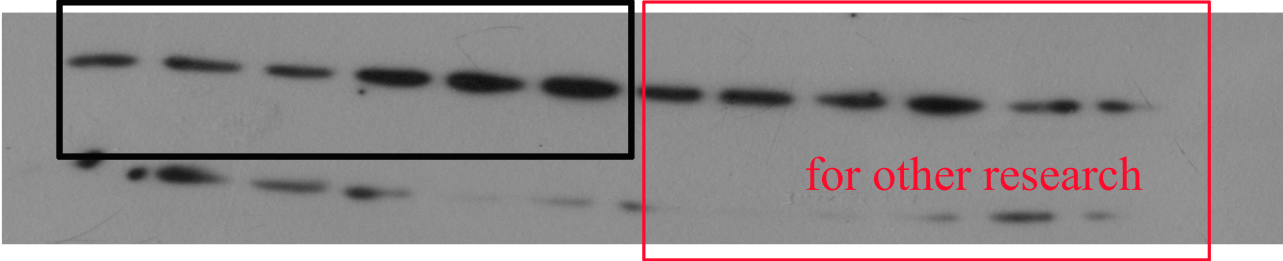

50 kDa

GAPDH

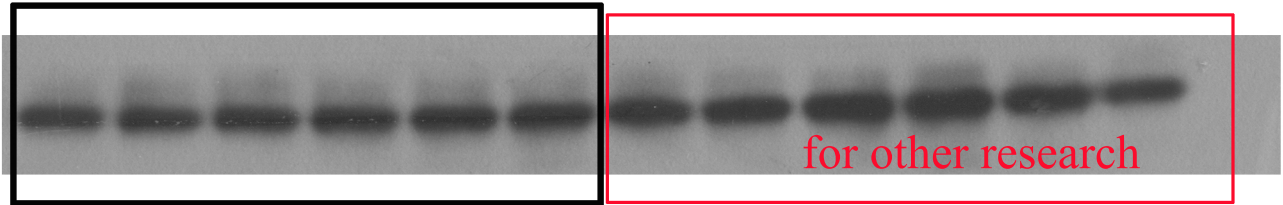

37 kDa

LXR $\beta$

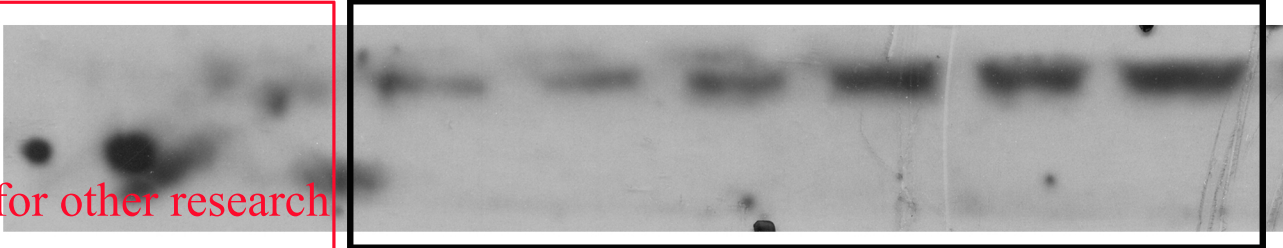

51 kDa

Precursor SREBP-1

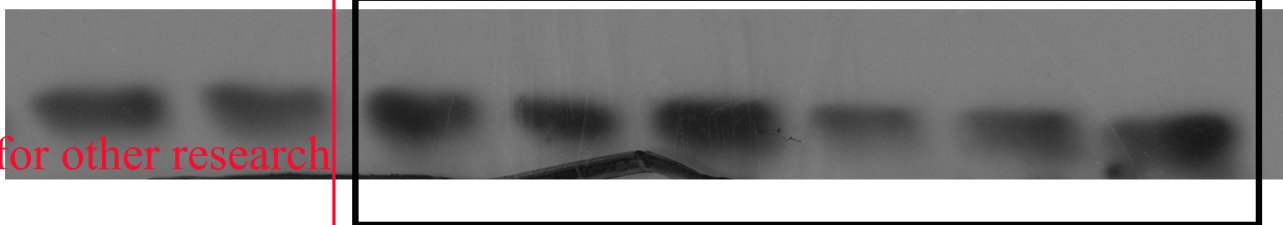

120 kDa

Mature SREBP-1

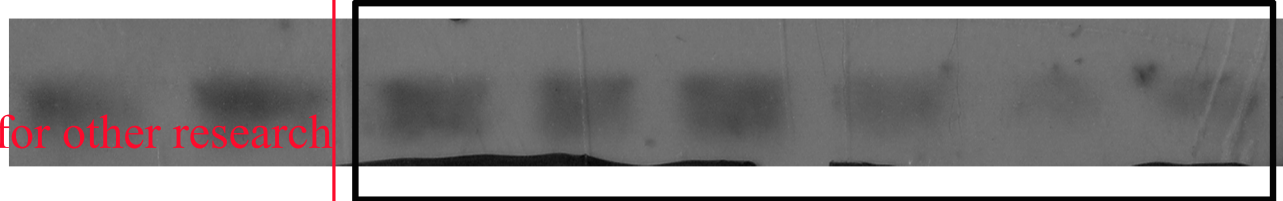

68 kDa

GAPDH

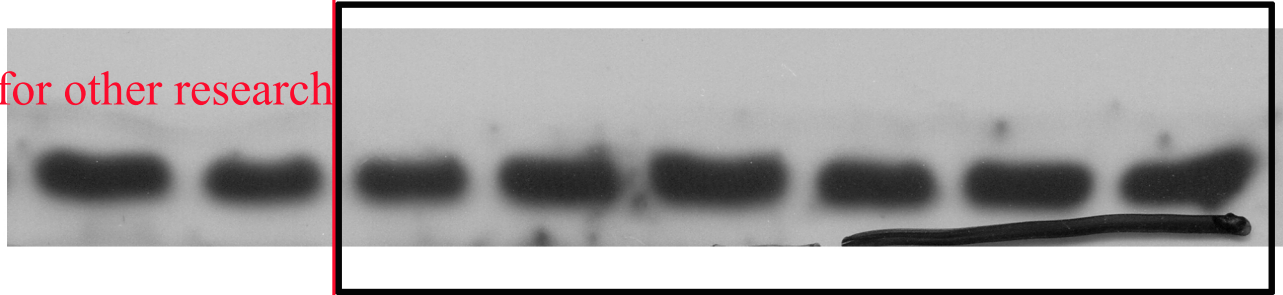

37 kDa

Supplement: FIGURE S1 — Original bands of Western blot for Figure 3B. [file Image_1.PDF]
